# On General Paralysis

**Published:** 1859-04-01

**Authors:** 


### Art. V.—ON GENERAL PARALYSIS.

The appearance of some recent memoirs, and the conclusion of a long-continued and interesting discussion on this important subject before the Medico-Psychological Society in Paris, occupying three entire sittings, seem to present a favourable opportunity for bringing before our readers the present state of our knowledge and our prospects as regards a disease which has only of late years been recognised as a distinct nosological entity, and upon which we are still far from absolute certainty in reference to any point connected with its history, except its extreme gravity and its almost certainly fatal result.

A preliminary idea of the nature of the affection of which we speak may be obtained by a glance over the various names by which it has been known. It has been called General Paralysis of the Insane, Paralytic Insanity, Incomplete and Progressive Paralysis, from its symptoms and its connexion with mental disorder; from its usual pathological characteristics it has been termed Slow or Chronic Meningitis, General Cortical Cerebritis, Diffuse Chronic Peri-encephalic Meningitis, and Meningo-Cerebritis. It is a disease which may be briefly said to be characterised by disorder of the intellectual and volitional powers, not always, or even generally, in strict proportion one to the other. The mental affection is usually of the *expansive* character, attended by exaggerated notions of the wealth, position, or personal qualifications of the subject, terminating in dementia. The physical disorder consists in a progressive weakness and uncertainty of action of the voluntary muscular system generally, in the majority of cases beginning with the muscles of the tongue and those connected with articulation, so that stammering is very often the first symptom of the invasion of this affection. In a patient who has evinced any tendency to mental derangement, there can be few symptoms of more serious import than this—a tendency to stammer, or a difficulty of pronunciation of certain words; it may be apprehended with a great amount of certainty that general paralysis is imminent, and that death, soon or late, will follow as the almost necessary result, let the physical health have been up to that time ever so good.

It may be expected that very soon the tongue and the muscles of the face will act irregularly and tremulously; and that this feebleness and uncertainty of function will extend to the entire locomotive system, sometimes attacking first the inferior and sometimes the superior extremities. The term "general paralysis" is, however, in some measure deceptive, as not indicative of the absolute phenomena of the disease in its progress. It is not until the latest stages that the paralysis becomes truly general, and no voluntary power is left, nor any action even of the sphincters. But it is in so far "*general*" as that it is not hemiplegia, nor paraplegia, nor *local* paralysis, differing from all these essentially, inasmuch as *all the muscles of the body* are indiscriminately liable to be attacked, and sooner or later are so affected.

It is rather singular that a disease so well marked in its phenomena, and so serious in its results, should only have attracted attention in so comparatively late a period of medical history. It is also more remarkable that although it was an English physician who first noticed it (John Haslam, in 1798), yet its systematic and scientific investigation has been left almost exclusively to our French brethren. Esquirol briefly commented upon it in 1816; Bayle first brought out a complete history of it as a meningitis in 1822; followed in 1826 by the admirable treatise on *The Paralysis of the Insane*, by Calmeil, a work which must always take classical rank amongst scientific investigations. Since this period many elaborate and excellent monographs have appeared in France, as well as countless shorter papers, making the natural history of this affection almost as complete as that of any well-known disease. This contrasts strongly with our own contributions to its literature: so far as we are aware, we have *no* separate work on the subject; and in systematic treatises on insanity, for the most part this is only mentioned in a passing way. Many of our hospitals for the insane simply refuse admission to patients affected with paralysis, as being incurable!

We have spoken of this as a highly important subject; and why? It is not numerically very prominent in the causes of death affecting our bills of mortality, although, perhaps, much more so than has hitherto been suspected; and yet there are reasons, and forcible ones, why it is deserving of much more attention than has as yet been accorded to it by English writers.

The physical causes of insanity are hitherto involved in great uncertainty. Some pathologists go so far as to assert that in a majority of cases of simple derangement of intellect, *no* morbid changes of the nervous centres can be detected, and that in a majority of the remainder such changes are too slight, too unimportant, and too undefined and unspecific to be considered as *causes*. Others aver that they have never examined the brain of a patient who has died insane, without detecting some morbid alteration of the brain or its membranes; and these authorities are certainly amongst those who have had opportunities, not by units or tens, but by hundreds of cases.* Again, others affirm

* Thus M. Ferrus, whose authority is unimpeachable, observes:—"I would say, without *any reserve*, that every time that I have opened the cranium of an insane person, I have *never* found the brain healthy, as compared with that of a man who had died without signs of intellectual disorder. I have *always* found thickening or adhesion of the membranes, hyperæmia, softening or induration of the grey or the white matter; just as I should say that I have never found in the normal condition the lungs and heart of a person who has been for a long time asthmatic. . . . Science is not yet sufficiently advanced for us to say, as regards the brain, that each alteration corresponds always to one and the same pathological phenomenon; that the *visible* and *palpable* changes are certainly in many cases deficient, but would, doubtless, be revealed were our means of investigation by the microscope and by chemical analysis more perfect. And, in addition to all this variety of opinion, it has to be taken into account that whatever may have been the proximate cause of the mental affection, *certain additional physical changes* must have taken place in order to cause death; and there is the superadded difficulty of deciding what has dethroned reason, and what has destroyed life. In short, we are still ignorant of what may cause mental aberration, and what is its essential material element; nor have we as yet obtained any certain starting-point, any irrefragable data whereon to found a clearly inductive system of research.

Such data, if we are not mistaken, may be ultimately afforded us by the disease to which we purpose to devote a few pages. For, although general paralysis* appears clearly proved to occur frequently as an isolated disease, and apart from all mental derangement, yet it seems also to be the normal termination of a certain form of insanity, and to be otherwise closely allied to it, as will appear hereafter. Now, in examining the nervous centres of those dead from general paralysis we often, or at least occasionally, observe on a superficial inspection the same absence of morbid changes as in insanity in its least complicated forms; and yet a more minute and careful investigation almost invariably detects morbid appearances, as uniform in their essential nature and distribution, as defined and specific in their significance, and as correspondent with the foregone physiological phenomena, as any observed in pericarditis, peritonitis, or any of the recognised inflammatory affections. They certainly require special methods of research for their detection in many of the less marked cases; but when found they are such as only occur in this and allied affections, and as occur all but invariably in these. There being then a tolerably uniform connexion between one phase of mental disorder and a certain form of physical derangement, manifested by paralysis of a most marked and distinctive kind—one, in fact, which cannot well be confounded with any other,—and this paralysis being dependent upon, or at least almost inseparably connected with, a clearly-defined pathological process and morbid change,—we may, by an analysis of such considerations, obtain a glimpse of some possible point of departure for attaining a knowledge of the material changes which accompany (if not but the case is the same as regards all the complex apparatus of organs, unless we reject the evidence of pathological anatomy. I do not think that the brain ought to be considered as placed in exceptional circumstances under these views."

* We adopt this designation only as the most convenient, and not from any conviction of its scientific merits; were it practicable in the present state of our knowledge, a pathological term would be preferable.

*cause*) psychical troubles. On these grounds we propose to investigate, carefully, the nature and phenomena of general paralysis; and in so doing we shall draw largely, where it seems expedient so to do, from the discussion above mentioned, in which MM. Parchappe, Pinel, Baillarger, Delasiauve, Ferrus, Belhomme, and Falret took a prominent part.

One of the principally contested points concerning this affection is, whether it is essentially connected or not with mental disorder, and what place it is entitled to occupy in a nosological classification. For it appears to occur sometimes entirely independent of mental affection; sometimes to supervene upon it; and sometimes to precede it by months, or perhaps years. In examining this question we shall follow the plan of M. Parchappe, and inquire into the causes, symptoms, seat, nature, and progress of the malady; as by so doing we shall attain a fuller comprehension of the subject than by any less systematic treatment. We consider it first as studied exclusively in connexion with mental disorder.

1. The *causes* of general paralysis are immediate and predisposing. The former are such in general as produce a prolonged over-excitement of the brain,—sensual excesses, especially the abuse of intoxicating drinks, over-feeding, sexual indulgence, and intellectual excesses accompanied by prolonged vigil. The predisposing causes may be considered to be such as belong to insanity and brain affections generally; the disease, moreover, attacks men much more frequently than women, and both chiefly between the ages of thirty and forty-five years.

2. The *symptoms* of general paralysis (of the insane) consist of an apyretic or non-febrile lesion of the intelligence, the sensibility, and voluntary motion. The intelligence is constantly affected from the commencement of the malady; sometimes only observed as a feebleness of memory or judgment; sometimes, and most frequently, as mania with the delirium of grandeur and power; sometimes as melancholia; and sometimes, again, as simple dementia from the outset, a condition into which all the previous forms have a tendency to merge as the disease makes progress. Whatever may be the original specific type of the mental affection, the faculties are observed to be enfeebled progressively, even during the excitement of the opening delirium; and this goes on until the last phase is characterised by the utter extinction of all intelligence.

The alteration of motility consists in progressive weakness of the voluntary movements, commencing by trembling of the muscles of the tongue and mouth, by a more or less marked difficulty in the pronunciation of words, and afterwards by hesitation in walking and uncertainty in standing, with trembling and weakness of the hands and arms. The loss of muscular power extends often, and especially in the latter stages, to the sphincters of the bladder and the anus. When arrived at the extreme, the loss of power condemns the sufferer to absolute immobility and mutism.

The loss of sensibility has not been studied with the same amount of care and attention as has been bestowed on that of motion and intelligence. Yet it is ascertained that the general sensibility is notably diminished, and the special tact or touch greatly deteriorated. The sight and hearing partake, in some measure, in the general dulness of sense.

3. The *seat* of the malady is in the cortical layer of the cerebral hemispheres. (We here give M. Parchappe's opinion and words.) Pathological anatomy furnishes a *positive* proof of this, by the constancy of the existence, in this layer, of alterations characteristic of an inflammatory condition, amongst which is prominent a softening of the cortical substance; and a *negative* proof by the absence, in some cases, of every other alteration, and particularly of all alteration in the meninges.

The anatomical proof is confirmed by the physiological considerations which place the seat of the affected functions, the intelligence, the voluntary motion, and the sensibility in the cortical layer of the cerebrum.

"I feel it necessary (says M. Parchappe) to meet beforehand some objections that may be made to this opinion. It is necessary in the first place to remark, that an error of diagnosis during life is not very difficult. There are cases of mania with volubility and stammering, which may be confounded with general paralysis, and which may be mistaken by the most skilful observers. There are cases of simple dementia, arrived at the extreme, with mutism and immobility; and cases of various maladies of the encephalon, with feebleness or abolition of the intelligence, difficulty of articulation and progression and impossibility of standing, which may simulate the general paralysis of the insane. In such cases, we must abstain from pronouncing, during life, upon the existence of the disease in question, if we wish to avoid deceiving ourselves, and compromising science by facts which are only apparently contradictory. . . . In all the cases of true *paralytic insanity* (*folie paralytique vraie*) in which I have been able to pronounce an accurate diagnosis during life (and these have amounted to 322), I have constantly and without exception satisfied myself of the existence of inflammatory softening to a greater or less extent of the cortical matter of the cerebral hemispheres. . . . Had I always confined myself to the ordinary methods of investigation, I should often have overlooked the existence of this characteristic alteration. The meninges were healthy, and detached themselves freely from the surface of the hemispheres. The cerebral surface was not altered in colour; in consistence it appeared even increased. The brain cut in slices appeared perfectly sound. But the handle of the scalpel lightly inserted into the grey matter would often indicate the existence of softening of the internal layer, by permitting that easy detachment or decortication which in the greater number of cases of softening is determined by simple traction of the membranes.... I believe that the cases of integrity of the cortical layer which have been brought forward are all explicable either by error of diagnosis during life, or by insufficiency in the methods of exploration after death."

The *nature* of these alterations, according to the same authority, is inflammatory. They have for their special characters, a tendency to extend simultaneously to the two hemispheres, especially to the anterior and middle lobes—to be associated almost constantly with inflammation of the meninges, and frequently with that of the grey matter of the cerebral ganglia, of the cerebellum and the spinal cord, with a granular alteration of the ventricular parietes and an induration of the white matter,—and finally, very frequently with atrophy of the convolutions.

M. Belhomme considers that the central ganglia of the brain are as much concerned in the pathological changes, as the cortical layer of the hemispheres. In seventeen observations, certain changes were constant,—viz., those involving the cortical layer, the tuber annulare, the cerebral peduncles, the walls of the third and fourth ventricles, and the medulla oblongata; all of which were either softened, or in some way pathologically altered: the membranes also were in all cases injected, thickened, and opaque. The other changes were :—

| | | |
|---|---|---|
| Adhesion of membranes to cerebrum . . . . | 14 | times. |
| Softening of medullary matter . . . . . . . | 12 | " |
| Induration . . . . . . . . . . . . . . . | 5 | " |
| Softening of the corpus callosum and septum lucidum . . . . . . . . . . . . . . . | 14 | " |
| Softening of tubercula quadrigemina . . . . | 13 | " |

From which observations it would appear to result that the degeneration of the brain in this disease is indeed general, and by no means confined to one part.

4. The *progress* of the development of this affection has certain characters which are special to it, as relating to the succession and connexion of the symptoms, and the termination of the disease.

Impairment of the memory and judgment is evident from the first, and continues always increasing until their entire abolition.

The alteration of motility only appears very sensibly after that of the intelligence. It may be absent at first, and continue so for some time, so as to cause doubts in the mind of even the most practised observers as to the nature of the malady for even some weeks. Most frequently it is manifested at first and chiefly in the articulation, and afterwards extends to the other voluntary movements, especially those concerned in walking and standing: though it does sometimes happen that these latter functions are seriously affected, whilst the speech is but little altered. It is not infrequent for the paralysis to appear simultaneously in the speech and the locomotive apparatus. Occasionally it is more pronounced on one side, so as to simulate hemiplegia.

A very important point to notice is the effect upon the muscular striæ of the iris, which share in the general feebleness, and act unequally, so as to produce want of accordance between the pupils.

The alteration of the sensibility, at first not very marked, follows in its development the impairment of the intellectual faculties; and it is only absolutely extinguished during the state of temporary congestion, or the state of permanent coma that precedes death.

In general, except in the congestive state, general paralysis is not accompanied by febrile symptoms. But this congestive state is one of the most characteristic symptoms of this malady; very frequently a cerebral congestion marks its outbreak, and thus the subsequent mental and physical phenomena are attributed too hastily to apoplexy. Habitually this congestion reappears many times in the course of the disease; and each time it leaves the patient with symptoms much aggravated.

These cerebral congestions are connected intimately with one of the most remarkable phenomena observed in general paralysis, *i. e.*, the almost complete remission of all the symptoms which occasionally is noticed. When these congestions do not reappear or are a long time between, the brain seems to become accustomed to its partially impaired condition, and to resume its functions almost normally—a state which continues until a new attack of congestion supervenes.

The softening of the cortical layer, and the other alterations of this substance and of the membranes, present during the first period, which may be called acute, all the characters assigned to the inflammatory condition, such as the rosy tint, hyperæmia, pointed injection, extravasations, morbid adhesions of the pia mater to the brain, and sometimes effusion between this membrane and the cerebral surface.

At a more advanced epoch, if death has not ensued during the congestive stage, we do not observe any hyperæmia. The cortical layer is then not only softened, but the convolutions are atrophied, and present a pale dirtyish grey or yellow colour; the membranes are thickened and opaque, and serum is effused, occupying the space left by the receding convolutions.

In the general paralysis of the insane there is a profoundly interesting connexion between the symptoms and the organic alterations, which deserves much and careful attention.

The disorder of the intelligence, under the form of mania or melancholia, coincides with the period during which the alterations of the cortical layer are only superficial or of small extent. The diminution of the intellectual faculties, as well as the paralysis of voluntary motion, seems connected most intimately with the depth and extent of the softening. The imperfection of articulation (Parchappe) is generally in relation with the extent and intensity to which the *anterior lobes* are affected.

Although this is a *general* affection of the cortical substance, it is not always and necessarily of the same intensity on both sides; and when there appears any hemiplegic tendency, it is generally observed that there is a greater amount of softening on the side of the brain opposite to that of the most affected limb.

One of the most special and distinctive, as well as most serious, characters of general paralysis is, that it *terminates constantly by death.* As we proceed, we may find some exceptions to this in the general paralysis occurring independent of mental disease; but most observers agree that when once general paralysis has appeared, *even slightly,* in an insane person, there may possibly be a remission of symptoms even for a considerable period, but there can be (or rather *has hitherto been*) no cure—death is imminent and certain, although at a very uncertain period. Perhaps the average duration may be from two to three years. It is very common before the close of life to observe gangrenous sloughs on all parts of the body that are exposed to pressure; the phenomena of life are merely vegetative for some time before dissolution.

M. Parchappe gives a very unhesitating opinion as to the nosological situation of this affection. He considers it essentially a species of mental unsoundness, and denominates it "*folie paralytique,*" for the following reasons:—

"It does not appear possible to ignore or break the strong bonds of connexion between this disease and simple insanity.

"The determining and predisposing causes are analogous.

"The disease frequently commences by intellectual disturbance, without any trace of paralysis; and for days or weeks, the patient who is about to be affected by general paralysis presents no symptoms but those of insanity. The paralytic phenomena are sometimes only developed after the insanity has lasted a long time. The disease has the same seat as insanity, viz., the cortical layer of the cerebral hemispheres. And although simple insanity is not characterised by any well-defined morbid appearances, yet such alterations as are frequently met with, and which some observers* even state to be always present, have the strongest analogy with those met with in general paralysis.

"Simple insanity may be considered to consist in a dynamic alteration of the nervous action merely. But this *dynamic* change becomes *plastic* in the last stage of dementia, by atrophy of the convolutions. And in my opinion, simple insanity, from being dynamic, becomes plastic, at the period when it becomes complicated with general paralysis.

"On the other hand, it does not appear easy to bring this disease under the head of inflammatory affections of the encephalon. It is apyretic: it is not marked at its outset by bilious vomitings, so habitual in meningitis, so frequent in encephalitis. It does not present the febrile and acute symptoms which characterise inflammations of the meninges, or the cerebral substance. True encephalitis is habitually *partial*, and occupies only one hemisphere; the paralysis is usually therefore of an hemiplegic order, and is often preceded and accompanied by contractions of the muscles. The inflammation also usually involves both grey and white matter.

"The progress, finally, of true encephalitis is essentially acute and rapid—it lasts only one or a few weeks, whilst general paralysis lasts months or years.

"Such are the reasons which have induced me to place general paralysis as to its nosological classification under the head of '*alienatio mentis*.'"

Such is a *résumé* of the opinions of M. Parchappe, one of the most constant and laborious investigators of this subject. There are, however, many points of controversy which, we think, will be brought forward most prominently and clearly by taking up the thread of the discussion above mentioned.

M. Delasiauve recognises the existence of the morbid changes above mentioned, but dissents from the conclusions. He views them as *consequences* of the progress of the disease, not as its *cause*. The disease *gives rise* to congestions, and the repeated congestions *produce* the softenings above described. In this he is equally opposed to the views of M. Parchappe, and to those of his much-regretted friend M. Bayle, who considered the essential nature of the disease to be chronic meningitis. He places the seat of the pathological process, whatever this may be, in the cerebral hemispheres, but does not consider it primarily inflammatory, but rather "*un de ces vices de nutrition dont le mystère, jusqu'à présent, n'a pas été éclairci.*" Acknowledging the congestions, he denies altogether their primitive *casual* influence, and considers them as produced by or subordinate to a secret pre-existing alteration (qy. dynamic?) in the brain, of which the specific influence is to trouble the circulation, and to produce *stasis* of the

* See the opinion of M. Ferrus above quoted.

blood in the encephalic vessels. He also considers that the remarkable intermissions so common in this disease are more easily explicable on this hypothesis than on that of a necessarily progressive organic change. He objects to the designation of "*folie paralytique,*" as involving an hypothesis, and inclines more strongly to that of "*paralysie générale.*" On the whole, his learned and thoughtful address was destructive rather than constructive.

M. Baillarger is of opinion that, under one general denomination, we have combined facts which are, at least apparently, very dissimilar. There are two principal groups which may be mentioned.

1. It is very common to see patients who have gradually fallen into *dementia* without presenting any of the symptoms of reaction or delirium, or in whom these have been extremely slight and subordinate. As this decay of the intelligence is established, we notice at first slight, and then more marked signs of paralysis; and shortly we have developed a paralytic dementia so marked, that none can mistake the phenomena.

2. By the side of this numerous group there is another which is yet more so, especially amongst men.

The patient begins by presenting the signs of an excitement more or less lively; he moves about perpetually, and cannot remain still for a moment. At the same time, he forms large projects, buys all manner of objects, gets angry when opposed, and cannot sleep. To this state succeeds a complete maniacal delirium, with predominance of ambitious ideas, and a special muscular agitation quite distinct from that of ordinary mania. At the same time, if we are in the habit of attentively observing such patients, we shall detect a *little hesitation in the pronunciation of certain words.*

Such are the two principal groups which are at present designated under one name, that of insane paralytics.

These symptoms are assuredly very much opposed. In the one case we see debility, inertia, and the slow extinction of the cerebral functions; in the other, force, violence, and an increase of physical and intellectual activity.

But, to reconcile these, it is said that one is the acute, the other the chronic state of the disease; that simple primitive dementia is rare, and, when it does occur, that the proper, legitimate, first, or excited stage is wanting; and thus the ambitious mania is but a period or a form of the general paralysis.

M. Baillarger doubts this interpretation and theory. The acute and chronic stages of a disease must present analogous symptoms and anatomical lesions:—only in the one case the progress will be rapid and acute, with phenomena of reaction; in the other it will be slow, and the reaction wanting. Thus in acute and chronic pleurisy we find these conditions united. But is it so with the ambitious mania and paralytic dementia? Is the one the chronic form of the other? We have seen how different are the symptoms—muscular and intellectual excitement contrasted with dementia and incomplete paralysis.

In regard to the anatomical lesions, these are not less contrasted. In patients who have died during acute ambitious mania, there is almost constantly congestion of the hemispheres, with increase of weight; whilst in paralytic dementia there is always diminution of volume and weight of the hemispheres, and serous effusion, which every one now admits to be *not* a cause of compression of the brain, but as merely poured out to fill up the void left by the retreating atrophied convolutions. Thus the average weight of the brains of patients who had died in the acute form of ambitious mania was about one-eighth more than the average of healthy brains; whilst those of patients who had suffered from paralytic dementia were about one-sixth *below* the average weight. This is strictly analogous to the results of the observations of M. Parchappe on the brains of those who have died during *simple* mania and dementia. In the former he always found hyperæmia, turgescence, and augmentation of weight of the brain; and in the latter, atrophy, with diminution of weight. In the present case, however, the contrast is still more marked: there is a greater increase of weight in ambitious than in simple mania; there is a greater diminution of weight in general paralysis than in simple dementia.

From all this M. Baillarger concludes, that in ambitious mania there is as yet but a congestive and strongly hyperæmic condition of the brain; whilst in paralytic dementia there is atrophy, accompanied by lesions of the cerebral substance of a very grave character, of which the principal are a granular condition of the grey matter, a special induration of the white matter of the convolutions (*sic*), and a *tendency to isolation* of the two substances of the brain.

Reasoning by symptoms and anatomical lesions, we ought to separate these two affections; and yet there is a reason why one might appear to be a phase or stage of the other, that is, that in a great majority of cases the paralytic dementia does succeed fatally to the ambitious mania. Some writers give this as the invariable termination. M. Esquirol states that in mania and monomania, if any symptoms, *however slight*, of paralysis be observed, we may predict with certainty that dementia will succeed, and that death will speedily terminate the disease. M. Baillarger admits that, without doubt, the ambitious maniacs who fall into dementia with paralysis are much more numerous than those of simple mania so terminating; but he believes that this is not the *absolutely necessary* termination. He adds that authors have related *a few* cases of recovery even after the patients had presented the gravest symptoms of paralysis; and that although these facts may be considered exceptional, they are doubtless of the highest significance. We must be content with the briefest abstract of a few of these cases.

" *Observation 1st.*—Mania for some months, with predominance of ambitious delirium; very marked symptoms of paralysis, with difficulty of walking and standing. *Stammering not present*, but some hesitation occasionally before a word. Formation of gangrenous sloughs, followed by recovery, which lasted twenty-five years.—(Related by M. Ferrus.)

" *Observation 2nd.*—M——, aged 41, had some insane relations, and became melancholic himself after some domestic trouble. He had some epileptic attacks; the speech became imperfect, and one arm weak. After his entry into the asylum, he was in continual agitation, and had the delirium of grandeur and riches fully developed. Pupils unequal, tongue furred, pulse accelerated. The sensibility became very much diminished, and the paralysis extended to the sphincters. He could not walk nor write his name: there appeared a great number of sloughs on the body, some apparently without cause, some where his agitation had bruised the skin. After attaining almost the lowest state of paralysis, he began to recover—as it is stated, not by means of therapeutic agency—and both his reason and his bodily powers returned. He was alive and well when this account was written, ten years afterwards.—(From the *American Journal of Medical Science.*)

" *Observation 3rd.*—General paralysis, apparently of the worst kind; symptoms of the last period. Acute œdema of one leg, with gangrene. Rapid recovery, which is known to have lasted six years.—(By M. Renaudin.)

" *Observation 4th.*—Acute mania, with tendency to ambitious ideas; feebleness of the memory; uncertain walk. Eruption of boils, with abundant suppuration. Embarrassment of speech; recovery. Duration of the malady, one year. Duration of the recovery, five years. Sudden death from cerebral hæmorrhage.

" The 5th case recovered after an abscess of the liver; the 6th after a purulent discharge from the ears; and the 8th after an amputation, followed by profuse suppuration."

In all these cases the paralysis was so far advanced as to be unmistakeable—the authorities from whom they are quoted are irrefragable. We must therefore admit the *possibility* of paralytic ambitious mania being cured, and therefore that it does not *of necessity* lapse into dementia. Death also sometimes takes place without the supervention of dementia. It is interesting to remark in how many of these cases of recovery the amendment seemed to date from the establishment of a suppurating surface, either spontaneous or artificial. Although the cases of true recovery are certainly rare, yet those of apparent restoration to a state of health, for a time more or less prolonged, are very frequent; and these remissions are amongst the most surprising of the phenomena of this affection.

M. Baillarger concludes, that ambitious mania presents different symptoms and different anatomical lesions to those of general paralysis with dementia; that these two maladies have an existence isolated the one from the other; that we cannot regard them as acute and chronic forms of one and the same affection; and that they ought to be considered apart, as are mania and dementia in their simple forms; being still more contrasted than these, both symptomatically and pathologically. From the very important part assigned to cerebral congestion in this disease, he proposes that it should be known as *mania congestive.*

It appears also not impossible, nor altogether improbable, that inasmuch as a considerable proportion of these cases of remission are discharged from the hospitals, and otherwise lost sight of, some of them, at least, *may be* instances of permanent recovery, similar to those above related; especially in those cases of amendment which have dated from critical discharges, or purulent evacuations, natural or artificial. With this possibility, and with the certainty that in some cases of ambitious mania with paralysis recoveries have taken place, we may indulge still the hope that further progress in our knowledge of the treatment may be attained; and at all events, we should never cease our efforts on the hypothesis that these cases are irremediable. It must, however, be acknowledged, that all authors are agreed on one point, viz., that they have never met with a case of *paralytic dementia* that has terminated otherwise than fatally.

Dr. Archambault, however, states that when he became attached to the "Charenton," he found there twelve or fifteen patients who had been in the establishment more than ten years, and who were stated, on their entry, to be affected with general paralysis; yet they only now presented very slight signs of the disorder. These and similar considerations would seem to indicate the necessity for a more guarded prognosis than is usually given in such cases.

We have hitherto considered general paralysis only as found united with mental disorder, and as inseparable from it, at some period of its history. There is, however, another aspect in which it may be viewed, of which the following is an outline drawn in great measure from an elaborate portrait by M. Pinel.

General paralysis is a special malady, characterised by lesion of motility, more or less extended, with a progressive tendency to become universal.

It exists either simply or complicated: in the first case, it is independent of every other affection, and there is no disorder of the intelligence; in the second, it is frequently united with some form of mental derangement.

The malady designated "general paralysis of the insane," or *folie paralytique,* is only a complication of general paralysis with insanity.

General paralysis may continue sometimes, although rarely, until death, with this complication with mental disorder. It is accompanied often with a loss of memory, especially for recent events, which is different from dementia.

Ordinarily, insanity supervenes, after a period more or less extended, to complicate the motor lesions. Most frequently the motor lesion first appears; sometimes the two classes of phenomena are simultaneous in their outbreak; less frequently, the paralysis is secondary.

Simple or uncomplicated general paralysis is not seen in asylums for the insane, for obvious reasons. Hence has arisen the idea that it is always connected with mental affection.

It may precede, accompany, or follow mania, monomania, melancholia, and especially dementia. The expansive (ambitious) delirium, often considered as a constant sign of general paralysis, is not infrequently altogether absent. With regard to the symptoms, progress, remissions and causes, nothing further need be advanced than what has been above stated.

"Moderate local bleedings, at the outset only, and when there are signs of congestion—issues at the base of the brain—repeated vesications over the scalp—general affusions, and revulsives from the intestines and extremities, are the principal means to be used. The prognosis is most unfavourable; yet there are well-authenticated instances of recovery."

"I am convinced that general paralysis is not a disease essentially appertaining to the insane—that it is not a form of insanity—that it does not necessarily and fatally induce it—that it is an affection entirely independent of this latter, so long as the alteration which produces it does not extend to that portion of the molecular structure of the brain which presides over intelligence; and I believe that this portion of the brain may escape lesion during the whole period of life." —(Pinel.)

It appears that although most cases are complicated with insanity at some period of the affection, and a considerable proportion of the remainder present a partial decay of the faculties, especially memory and judgment, still far short of dementia; yet certain of the authorities, amongst whom we find Rostan, Guislain, and Pinel, are convinced that there are cases of general paralysis of the most marked kind which from first to last show no affection whatever of the mind. M. Rostan even states that five out of six of the cases of general paralysis which he has attended were not insane patients; and MM. Guislain and Pinel both agree that they have seen patients die of general paralysis who had never evinced the least disturbance or enfeeblement of the intellect. These authorities all agree, for the most part, that general paralysis most frequently induces ultimately some mental disturbance; but are of opinion that it does so, not from any original and essential connexion with insanity, but just as epilepsy, apoplexy, chorea, and convulsions act, without ever having been considered special forms of insanity.

The question of the essential nature of general paralysis has perhaps been complicated, more than by any other consideration, by that of the variety of manners in which it makes its first appearance. We will briefly sketch these on the plan of M. Falret, before attempting to give a concise statement of our own views on the whole subject.

General paralysis presents itself originally in four forms, two of which are marked by predominance of somatic disorder, and two by that of the mental phenomena. It is important to note this, because it has been perhaps too generally considered that the expansive delirium was essential to its diagnosis in the first instance. This is, in reality, its most frequent form, but by no means the only one. There is a fortuitous reason why this form has been stated to be the essential characteristic; and that is, that in the other modes of development, the appearance of the disease is so insidious, that its early periods have passed before the patient comes under special notice, or is placed in an asylum; but the ambitious or expansive delirium is so marked in its characters, that the subject is placed under surveillance at once; and so it happens that almost all the cases of general paralysis which are seen early bear this type.

1. The *paralytic variety.*—This is the most insidious of all the forms in which this fearful disease appears, and has perhaps given rise to more contest as to its nature than any other. For some time the only disorder appears, to the ordinary observer, to be one of the motor functions exclusively. The patient himself perceives that his actions become irregular, trembling, and wanting in precision. He lets fall objects which he holds in his hands, and cannot perform any acts which require delicacy of manipulation; writing, drawing, or playing on any instrument, becomes difficult. He stumbles against the slightest obstacle, and walks with a jerk: he is more readily fatigued than formerly, and all his motions lack co-ordination. All this, which comes on very slowly and almost imperceptibly, is accompanied from the outset by a peculiar hesitation and embarrassment of the articulation, or stammering. Ordinarily there is slight pain in the head, and dizziness—an unequal dilatation of the pupils, and not unfrequently genital impotence, with occasional incontinence of urine.

During this period the patient is conscious of his failing strength, and troubles himself about it. He appears at first to enjoy the full exercise of his faculties; but although in some rare cases it is not possible to detect any intellectual weakness, in general the practised observer will recognise some indication of this. There is an undefined alteration of character or disposition —a mobility of temperament that scarcely admits of description —the performance of odd, bizarre acts that pass for eccentricity— a tendency to make mistakes in his usual employment—a slight weakness of memory for recent events—and, what is the most serious symptom of all, a combination of a marked hypochondriac tendency, with a general feeling and expression of satisfaction and pleasure, the sure forerunner to an outbreak of the expansive or ambitious maniacal delirium, which is not then long delayed.

2. The *congestive variety.*—In this form the physical symptoms again predominate over the psychical. The predominant character is one of transient and recurrent congestion of the brain, with or without loss of consciousness; ordinarily slight, though sometimes very severe, simulating apoplexy or epilepsy; and giving rise to the opinion that the subsequent paralysis has been caused by one of these affections. This form of congestion is distinguished from the ordinary apoplectic form, by leaving more serious and persistent traces in the moral and physical nature. The speech remains for a long time disordered, the movements of the limbs become difficult, and sometimes there is even incomplete hemiplegia, which diminishes and disappears slowly, to return perhaps after the next congestion. The intelligence is affected after each attack, as well as the motor powers; but if there be any considerable interval, both orders of phenomena partially disappear, and the patient is restored to a comparatively healthy condition—*apparently.* Immediately after the attack we may detect a considerable feebleness of memory and the other faculties; but in time the intellect appears to resume its activity. After several attacks, however, both the physical and intellectual nature is found to be unmistakeably deteriorated, and dementia, accompanied by various forms of delirium, supervenes. In short, like the last form, the malady assumes the ordinary aspect of "general paralysis of the insane."

3. *Melancholic variety.*—In this form the psychical disorder first attracts attention. It is only in comparatively rare instances that the physician sees the first stage of the malady; but in inquiring into the history of paralytics, he will not unfrequently find that the first phenomenon that attracted attention was a state of marked moral and physical feebleness or depression, with lowness of spirits, and incapacity, real or supposed, for any form of action; presenting every appearance of hypochondriacal melancholia. Even at this period, there may sometimes be detected slight disorders of motility, as stammering or hesitation in speech, or feebleness and trembling of the limbs. But these symptoms are overlooked, the melancholia absorbing the whole attention. This melancholic period may be very short, or it may have lasted some time; but in all cases that are paralytic it disappears gradually, to give place to the normal condition. But this is very transitory; almost as soon as the signs of melancholia begin to disappear, the attentive observer may detect frequently the symptoms of an opposed condition. The patient experiences a sensation of exaggerated comfort—he was never so well in his life, body or mind; he begins to be unnaturally active, moving incessantly; he forms projects of which he would never have thought before—perhaps not absurd, not altogether out of accordance with his fortune or profession, but still quite opposed to his previous habits and tastes. He then either passes gradually into the true expansive delirium, or a fierce mania with predominance of ambitious ideas breaks out suddenly, after which his history is the same as that of the next variety.

4. *Expansive variety.*—This is the most frequent form of the *début* of general paralysis; and from the marked character of the symptoms clearly requiring immediate attention, it is more frequently observed in its earlier stages than any of the others; and on this account has been erroneously supposed absolutely essential to the character of general paralysis. Those paralytics who present themselves from the outset with the expansive form of delirium, have been ordinarily men of active minds and habits, enterprising, rash, and generally of irritable, violent character, with much generosity. The expansive delirium is an exaggeration of this character; and so gradually does it sometimes sweep on, that the moment of actual outbreak is difficult of detection. The patient appears at first to be simply *more* active, *more* rash, *more* irritable, and by fits and starts more generous and lavish in his expenditure than before. By and bye all this passes clearly beyond the normal state. The subject is in perpetual motion, takes no rest, cannot sleep, feels an exaggerated sense of power and general *bien-être,* and conceives projects of the most stupendous character, that must be at once carried out, all of which, if not quite irrational, are opposed utterly to his previous habits and tastes. He adopts excesses which are not habitual to him, passes an irregular and disorderly life, and commits acts which greatly astonish his friends, such as undressing in society, sleeping in the fields and out of his own house, and perhaps committing petty thefts without any motive. He is possessed of boundless wealth or power, with which he will perform the most unheard-of deeds. He will give away his property; and if he has none, he will profess to do so. He is endowed with supernatural attributes, he is God's vicegerent upon earth—he is some mighty potentate. Acting upon some of these convictions, he commits an offence against society, himself, or property, which demands his seclusion, when the true nature of the case is readily recognised.

These four forms in which the disease manifests itself are so diverse as to lead many to demand its separation into two or more distinct affections. But the diversity does not end here; the progress of the paralysis is marked by many varieties, the most obvious of which is the acute and the chronic form, the latter being by much the most frequent. Some subjects remain in a state of calm dementia for many years, or even until death; whilst others are in a constant state of maniacal agitation to the end. Some progress from bad to worse constantly; others present remarkable intermissions, and apparent temporary restorations to health. In some the special symptoms of paralysis are strongly marked during the whole course of the disease; whilst in others they are so slight during most of the period, as to lead to much doubt on the nature of the affection. The state of the intelligence is equally variable; in some presenting merely enfeeblement of the faculties, scarcely amounting to insanity—in others manifesting the most grotesque and violent delirious conceptions. In many cases the delirium is of the expansive order, treating of riches, grandeur, and power—in others this form is entirely wanting, and its place is assumed by a low melancholic or hypochondriacal derangement. Yet, with all these differences, the various forms of the affection present so many points of relation or identity, and all have so marked a tendency to pass into one well-defined condition, characterised by physical and moral feebleness and vacillation—symptoms *a* and *b* give place so promptly and frequently to symptoms *c* and *d*, and *vice versa*, that we can easily perceive the unity of the pathological process through all the diversities of external manifestation. Nor is this the only disease which is recognised as *one*, in spite of the greatest varieties in its progress. We need enter into no prolonged illustration, but merely mention *phthisis pulmonalis;* the most cursory glance over the phenomena and relations of which affection will show us greater diversities even than those met with in our present investigation, with an almost equally uniform termination in death.

We may now attempt to indicate what appears to ourselves to be the order of the phenomena and the relations of this interesting affection. Under the influence of certain causes, of such a nature as to produce prolonged over-excitement of the brain, amongst which stand out in bold relief the abuse of intoxicating drinks and sexual excesses, acting especially upon constitutions hereditarily or otherwise predisposed to degenerative affections—under the influence (we say) of such determining and predisposing causes, the nervous energy is deteriorated and exhausted; and this result, at first *dynamic* merely, is succeeded, in accordance with the general law of all organs long *functionally* affected, by a change in the tissue itself. This change is anatomically betrayed in the earlier stages of the disease by signs of considerable congestion, attended by increased weight of the cerebrum; and in the later stages, by atrophy of the convolutions, with comparative anæmia and loss of weight. On both these we have a few remarks to offer.

We conceive that the first development of the congestion is chiefly statical, and is a result of the want of that peculiar organic nervous energy which exerts so remarkable an influence over the capillary circulation in all parts of the body; aided by the continual distension of the vessels during the functional excitement of the organ. At times this becomes active, owing to the effort made by the arterial system to relieve itself of the oppressive weight in front. This effort seems to be occasionally successful, and to be followed by relief; at other times it only aggravates the mischief. This congestion appears, though not primitively inflammatory, (and here we venture to differ from Bayle, Parchappe, and other great authorities,) to assume from time to time the form of a low chronic inflammatory action, which leaves its traces in thickening and opacity of the membranes, and certain other changes in the brain-substance itself, above described.

With regard to the atrophy and softening of the convolutions, and the effusion of serum on the surface of the brain and within the ventricles, we are not inclined to attribute them to inflammatory action in any degree, but rather to a defect of nutrition of the organ, sometimes primitive, and sometimes secondary to the obstruction to the circulation, coincident with the congestion previously mentioned—a degeneration of tissue which is met with very frequently in other organs without any evidence of previous inflammation. The serum seems by general consent to be recognised as merely poured out to supply the place of the retreating organ. It must be remarked that this degeneration chiefly affects the grey matter in which the dynamic force of the organ is generally supposed to reside.

Now it is necessary to inquire how far these views coincide with the symptoms during life, and what are the marks by which these pathological changes betray themselves in the course of their development.

The data both of physiology and pathology lead us to the conclusion, that although we are not yet able to point out the precise seat of the various functions of the brain, we must recognise a *different* seat for intelligence, volition, sensation, and voluntary motion. (We designedly separate between volition and voluntary motion, the functions being essentially distinct; it is not here the place to discuss this matter; but the fact as here stated is abundantly demonstrable, from the frequent phenomena of the contrast of the earnestness of the will, with the imperfection of muscular performance.) Although, then, the special character of this peculiar degeneration is to spread and attack indiscriminately the whole of the organ, but especially the grey matter; yet, judging from the phenomena of the disease, it appears not to expend its violence upon all parts equally at first; and according as the mal-nutrition or congestion affects first those layers or sections of the brain which preside over the different functions above mentioned, so the order of the phenomena is either somato-psychical, or psycho-somatic—either the intellectual disturbance precedes and predominates over that of the muscular system, or the symptoms of paralysis precede those of insanity; and thus also we have two distinct orders of symptoms, one connected with the physical, and the other with the moral nature—a somatic and a psychical general paralysis.

We believe that an attentive consideration of the phenomena of drunkenness will tend to throw much light upon the symptoms attendant upon the early or congestive stage, and likewise to illustrate and explain the striking varieties observed in the outbreak of this affection. There is the most accurate resemblance between these two, only that the one is transitory, whilst the other is progressive and permanent. There is probably the same congestion—there is the same characteristic stammering or difficulty in articulating certain words—the same weakness, uncertainty or trembling of the arms and legs, and general muscular system,—the same mental disturbance.

Now, according to the varying constitution of the subject, the excitement of intoxicating liquors appears first in various forms. One man will betray his intoxication merely by increased sententiousness, moroseness, pomposity, or irritability—in another the first symptom will be an expansive gaiety, and a manifestation of exaggerated universal philanthropy—another will be merely affected in his speech, the intelligence remaining intact—and again another will have his ideas so confused, and yet his consciousness so perfect, that he will not attempt conversation which he knows he cannot accomplish. In other cases it will be the locomotive system that is affected; the legs are unable to support the body, or to perform co-ordinate movements—or the hands are not competent to accomplish those delicate manipulations to which they are accustomed.

Strictly analogous are the phenomena of the *début* of general paralysis—all probably due to one and the same cause, congestion affecting some portion or portions of the nervous tissue. In the majority of cases, the congestive stage corresponds with the expansive form of delirium, just as in intoxication the first result is most frequently excitement of mind and body; but in other cases it produces depression and melancholia, and in others again it affects primarily the muscular system only. In most instances all the phenomena, both of mind and body, occur at some period or other; but the order in which they occur is susceptible of every possible variation.

By these considerations it appears that we may explain all the varieties of the disease on rational principles, and also reconcile all the conflicting views which have been advanced as to its nature.

The degeneration of tissue may attack first that part of the nervous tissue which presides over voluntary motion; and the symptoms of paralysis will then be predominant, or even exclusively present. We shall then have the simple, uncomplicated general paralysis mentioned by Rostan, Pinel, and others.

It may first attack that part which is the seat of the intellect, whichever that may be; and only affecting very lightly and in a secondary manner the locomotive part, we shall have insanity in which paralysis only appears late as a complication.

When the disease attacks simultaneously the whole of the dynamic apparatus of the brain, the somatic and psychical phenomena appear at once, and we have the "*general paralysis of the insane*"—the form of disease which has been most studied, and to which attention was first directed, and through which the whole subject has been submitted to investigation.

Taking this view of the matter, we shall now proceed briefly to sketch the phenomena of the disease in its commencement, progress, and termination; and as it seems a matter of uncertainty whether it is most frequent for the paralysis or the insanity to appear earliest, we shall review the two orders of symptoms separately.

The phenomena of *uncomplicated* general paralysis are *somatic:* the tongue, the lips, the limbs, present the first signs of the malady. These signs are a hesitation or embarrassment in the pronunciation of certain words or letters, and sometimes an impossibility of articulation—a spasmodic trembling of the lips and tongue, and a kind of vermicular action of the latter—a feebleness and trembling of the hands—an uncertain gait, lacking coordination of movement in the legs; and a difficulty in preserving the equilibrium—trailing of one or both feet—curvature of direction in walking, and involuntary flexion of the femoro-tibial articulation.

"When these symptoms (says Pinel) supervene after a cerebral congestion, with or without loss of consciousness; or when they manifest themselves slowly and gradually; when they are very perceptible one day, and disappear to recur the next; when to these phenomena are added augmentation of volume and clamminess of the tongue, roughness or feebleness of voice, amounting sometimes to aphonia, heaviness of the features, dulness of eye, insomnia, continual movement, dilatation of one pupil, flickering of the eyelids, and partial anæsthesia of the, skin, &c. &c., we may affirm almost positively the existence of general paralysis."

It is customary with authors to describe three stages in the progress of general paralysis; but there does not appear to be any practical benefit derivable from this course, and it is not by any means clearly ascertainable that there are three defined periods in the natural history of the affection, nor any marks by which one can be positively distinguished from the other. For instance, the loss of power in the sphincters and the formation of gangrenous sloughs may occur in what is considered the first stage; whilst retention of urine and most obstinate constipation may extend to the third or last stage. The second stage, as described, is sometimes marked by transitory excitement, by grinding of the teeth, strabismus, difficulty of swallowing and mastication, with enormous gluttony and general increase of the paralytic affection. The third stage is marked by ptosis, and dilated immoveable pupils; the eyes are fixed, the patient appears to hear nothing, and emits vague, confused sounds—sensibility is almost completely abolished. The fatal termination is frequently heralded by intractable diarrhœa, infiltration of the extremities, and gangrenous eschars upon the parts subjected to pressure. The bones necrose; and gangrene of the lungs and bloody tumours in the ears have been often noticed. Finally, the external surface becomes cadaverous, there is progressive emaciation, the mouth is loaded with sordes, the breath is fœtid, the respiration slow, the pulse feeble and intermittent, and death closes the scene.

After the full details that have been given above, it seems scarcely necessary to enter again into any history of the mental phenomena which accompany or supervene upon those of the physical order. The loss of memory for recent events is certainly the most characteristic symptom, and is almost invariably present, to some extent at least, even in those cases which are considered to be simple or uncomplicated. There is also very generally remarked an enfeeblement of the affective faculties—a growing indifference to the claims of friendship or relationship. Whatever may be the form of mental derangement that first presents itself, there is one general tendency, which is to resolve itself into *dementia,* which sooner or later almost always supervenes, unless death takes place early in the course of the disease, which may occur from apoplexy, or other apparently accidental causes.

The diagnosis may be doubtful in the outset; but very soon the hesitation of speech, the uncertain gait, and the loss of memory will make the nature of the disease certain. Delirium tremens may at first be occasionally mistaken for approaching general paralysis, but a few days will inevitably rectify this judgment. From all the varieties of spinal paralysis, this affection is distinguished by the embarrassment of speech and the lesion of intelligence, both which are entirely wanting in spinal disorders. The distinction from cerebral hæmmorrhage is obvious. Two other points connected with diagnosis merit a very brief attention. It has been said that there are many patients presenting the delirium of riches and grandeur in asylums who never become paralytic. And this is true; but an attentive observation will detect an important difference in the nature of the aberration. The true *expansive* delirium is *coherent,* granting the fundamental position; and this is almost invariably connected with general paralysis; whilst the mere maniac who calls himself a king, or emperor, or general, has nothing plausible in word or idea whereby to support his claims. This has been pointed out by M. Falret.

The second point is more interesting. M. Brierre de Boismont has shown that there are two divisions of general paralysis—one connected with, the other independent of, insanity. In the former class the muscular tissue preserves its irritability, as shown by local galvanism; in the scond class this is entirely lost, when the paralysis is perfect.

It is to be regretted that, whilst the natural history of this affection is so complete, nothing satisfactory can be said concerning its treatment. It is true that there are records of recoveries from the earlier or congestive stages, and even from conditions where the paralysis was so far advanced as to appear utterly hopeless. But in nearly all, if not all, of these cases, treatment seemed to be but little concerned in the result; the efforts of nature alone appeared to be effective. A hint for future investigation seems to be afforded by the almost uniform occurrence of a copious purulent discharge from some part of the system previous to amendment; but hitherto any attempts to imitate this process artificially have been unattended by success. One thing, however, these recoveries teach us, and that is, not to be in too great haste to relinquish our endeavours after a cure, nor to view as absolutely hopeless even the most unpromising cases.

We have given but few illustrative cases in this sketch, as from time to time many have appeared in this journal, and especially during the past year. The completion of this part, and the discussion of many other interesting points connected with general paralysis, must be deferred to some future opportunity.

---

### Art. VI.—ON THE MORAL THERAPEUTICS OF LONDON.

